# We Are What, When, And How We Eat: The Evolutionary Impact of Dietary Shifts on Physical and Cognitive Development, Health, and Disease

**DOI:** 10.1016/j.advnut.2024.100280

**Published:** 2024-07-25

**Authors:** Nicola Luigi Bragazzi, Daniele Del Rio, Emeran A Mayer, Pedro Mena

**Affiliations:** 1 Human Nutrition Unit (HNU), Department of Food and Drugs, University of Parma, Parma, Italy; 2 Goodman-Luskin Microbiome Center, David Geffen School of Medicine, University of California, Los Angeles, CA, United States; 3 G. Oppenheimer Center for Neurobiology of Stress and Resilience, Vatche and Tamar Manoukian Division of Digestive Diseases, David Geffen School of Medicine, University of California, Los Angeles, CA, United States

**Keywords:** human evolution, hominoids, hominins, and human ancestors, paleogenome, paleomicrobiome, paleometabolome, dietary habits

## Abstract

“We are what, when, and how we eat”: the evolution of human dietary habits mirrors the evolution of humans themselves. Key developments in human history, such as the advent of stone tool technology, the shift to a meat-based diet, control of fire, advancements in cooking and fermentation techniques, and the domestication of plants and animals, have significantly influenced human anatomical, physiological, social, cognitive, and behavioral changes. Advancements in scientific methods, such as the analysis of microfossils like starch granules, plant-derived phytoliths, and coprolites, have yielded unprecedented insights into past diets. Nonetheless, the isolation of ancient food matrices remains analytically challenging. Future technological breakthroughs and a more comprehensive integration of paleogenomics, paleoproteomics, paleoglycomics, and paleometabolomics will enable a more nuanced understanding of early human ancestors’ diets, which holds the potential to guide contemporary dietary recommendations and tackle modern health challenges, with far-reaching implications for human well-being, and ecological impact on the planet.

**Statements of Significance**

The present review provides novel and comprehensive insights into the evolutionary impact of dietary shifts on human development by integrating advanced scientific methods such as paleogenomics, paleoproteomics, paleoglycomics, and paleometabolomics, offering an unprecedented understanding of early human diets and their implications for contemporary health. It shows how the evolution of human dietary habits, marked by significant technological and environmental shifts, has profoundly influenced our physical, cognitive, and social development, providing crucial insights into modern health challenges and potential dietary recommendations.

## Introduction

The history of dietary habits is the history of humankind: hominins’ evolution has paralleled major shifts such as the introduction of stone tool technology, meat-eating, control of fire, cooking, fermentation, plant and animal domestication, which, in turn, have been associated with anatomical, physiological, sociocultural, and behavioral changes [[1], [2], [3]].

Hominins experienced an increase in body size and brain volume coupled with a profound functional reorganization of the cerebral anatomy, reductions in gut size, and modifications in tooth shape and structure—Aiello–Wheeler’s “expensive tissue hypothesis” [4]. Even though the link among encephalization, body mass, dentition trends, modifications of intestinal anatomy and function is not entirely understood [5,6], this theory posits a tradeoff between the bioenergetic costs of the brain and other energetically expensive organs, primarily the digestive system, and states that the increased metabolic requirements of larger brains would have been offset by a corresponding decrease in the gut size. To the contrary, some empirical evidence has shown no negative correlation between brain and digestive tract sizes, emphasizing the importance of considering alternative energy tradeoffs, such as the relationship between brain size and fat storage, in the evolution of mammalian brain [6]. Other factors could have, indeed, led to the development of large brains in humans: dietary shifts could have influenced the structure of social groups and cooperation dynamics. For instance, the sharing of high-value foods could have fostered reciprocal relationships within increasingly complex communities, contributing to the development of advanced cognitive skills [7]. Another mediating factor could be the human microbiome: dietary shifts along hominins’ evolution likely shaped gut microbiota composition and functionality as well [8].

Besides contributing to the maintenance of human health, dietary shifts have contributed to shaping the human pathophysiological landscape with the introduction of foodborne and diet-related diseases. There is, indeed, a complex interplay between disease ecology [9,10] and dietary ecology [11]: within the environment, the intricate food web, dietary preferences, trophic levels, feeding and foraging behavior, ecological interactions, the biological makeup of the individuals, their microbiome, evolutionary adaptations, and circulating pathogens mutually influence one another.

Diet imposes constraints on an array of “characteristics ranging from body size and life history strategies to geographic range, habitat choice, and social behavior” [11]. These constraints are multidirectional: diet reflects an organism’s energy intake and nutritional requirements, with different diets influencing growth rates, reproductive strategies, and overall life history traits [[12], [13], [14]]. Further, an organism’s diet can influence habitat preferences and geographic distribution, as well as shape the social structure and behavior of groups [15]. Dietary choices can have multiple effects on ecosystems, with changes in the abundance of certain foods influencing the interactions between species at different trophic levels—Persson’s “trophic cascades hypothesis” [16].

Other factors have contributed as well, including climate change—global rapid cooling and drying at the end of the Middle Miocene, increased seasonality in Europe and Africa, and contrasted year-round seasons—the expansion of grassland and savannah ecosystems, resulting in ecologically diverse scenarios exhibiting greater spatiotemporal heterogeneity and instability [17,18].

Although the impact of these changes on human physical and cognitive development, health, and disease has been highly explored, the contribution of dietary shifts has been relatively overlooked or limited to small evolutionary timescales.

In the following sections, we will review how the history of dietary habits is intertwined with the biological, cultural, and social history of humans: hominins’ evolution is, indeed, closely connected to changes in dietary preferences and behaviors, and it is a complex history of coevolution, cascading, multifaceted, and interacting events.

## Early Hominoid and Hominin Diets: Evolutionary Adaptations to Plant Consumption

Earliest hominoids pursued frugivorous habits, whereas subsequent hominins likely had diets primarily composed of plant-based foods such as fruits, leaves, and some tough plant materials [19,20], including large seeds and starch-rich underground storage organs, such as bulbs and corm [21]. Their molars and premolars had, indeed, relatively large, thick enamel, indicating a diet that required significant chewing. However, they were not specialists [[22], [23], [24]] but rather highly opportunistic and also consumed high-quality animal foods, as shown by radiocarbon dating and stable carbon isotope analysis, advanced biogeochemical tools, and microwear/mesowear data (Table 1).

**TABLE 1:** Techniques that can be leveraged to reconstruct paleodiets

Analytical techniques		What information they provide	What information they do not provide
Craniofacial and dental analysis	Dental morphology and geometric morphometrics (qualitative and quantitative observation of the shapes of molar cusps and mandible morphology; recent developments incorporate Artificial Intelligence/Machine Learning, including support vector machine algorithms)	They provide information about functional and morphological adaptations to a leaf- or fruit-based diet or to a more general one	Although they provide information about broad dietary categories, they cannot provide information about specific foods consumed
	Dental microwear and mesowear texture analysis (DMTA)	They provide information about broad dietary patterns in terms of fracture-resistance properties of the foods eaten in the weeks before death	They do not provide or provide little and insufficient information about morphological adaptations
	Finite element modeling and biomechanical modeling studies of craniofacial strain	They provide information about the mechanical basis of form-function relationship (i.e., chewing biomechanics)	They do not provide information about the particular type of food consumed and their frequency
	Enamel chip and crack analyses	They provide information about diets and biomechanics	They do not provide information about specific foods consumed
Biogeochemistry	Stable nitrogen and carbon isotopic analyses (including stable isotope ratios, like ^18^O/^16^O) from bone collagen (carbon and nitrogen) and enamel carbonate (carbon)	They provide information about broad dietary patterns	They do not provide or provide little and insufficient information about morphological adaptations
Zooarcheology	Faunal analysis, taxonomic identification, butchery marks and cutmarks, bone modifications and breakage patterns, zooarcheology assemblage composition analysis, and animal microwear/mesowear analyses	They can provide information about dietary intakes, relative importance of food sources, and hunting and subsistence strategies	They cannot provide detailed information about specific foods consumed, food preparation, and cooking methods
Archaeobotany (paleoethnobotany)	Carpological analysis, including analysis of microbotanical remains (starch granules, phytoliths, and starch-rich underground storage organs, such as bulbs and corms)	They can provide information about dietary intakes, cooking and food processing, seasonality and agricultural practices, food storage, and preservation	They cannot provide information about processing and consumption patterns
Molecular analysis (paleogenomics, paleoproteomics, paleoglycomics, paleometabolomics, and paleometagenomics)	Genomic study of DNA extracted from dental calculus and evolutionary scans (inter- and intraspecific)	They can provide information about dietary components consumed, domesticated plants and animals, genetic variants for dietary adaptation, human-animal and human-plant interactions	They cannot provide information about dietary intake, processing and cooking (even though they can show which adaptive mechanisms can arise after the introduction of specific processing and cooking strategies)
	Metabolomic, genomic and shotgun metagenomic study of coprolites and paleofeces	They can provide information about dietary components, microbial community composition, fermentation processes, and the impact of diet on health status	They cannot provide detailed information about specific foods consumed

To survive, hominins developed strategies and abilities to make use of food not necessarily of high nutritional value (the so-called “fallback food”) [25,26], which may have become important diet components in times of food scarcity, exerting strong selective pressures. Indeed, it is not by chance that there exists an overlap between diet-related adaptations and those induced by climate, seasonality, and environment.

The adaptations to plant-based dietary habits [27] show moderate-high inheritability, around 49%, as found in a United Kingdom sample investigating the genetics of choice of fruit and vegetable sources [28,29]. Evolutionary adaptations to plant-based foods seem to have selected a set of genes, including one coding for salivary α-amylase (*AMY1*), which can be found across various taxonomic kingdoms and has a long evolutionary history marked by gene duplications leading to multiple paralogs. *AMY1* exhibits copy number (CN) variation (CNV) in numerous species, and among primates, humans stand out with a higher average count of *AMY1* copies [30]. This variability has been linked to the amount of dietary starch: “traditional populations” consuming high-starch diets have higher average *AMY1* CN than populations relying on few starchy foods [31]. Consequently, the increased CN of *AMY1* in humans may be the result of natural selection favoring more effective starch digestion [30].

In modern humans, lower *AMY1-*CN and serum amylase concentrations are associated with higher BMI, waist circumference, insulin resistance, an increased rate of cardiovascular disease and metabolic syndrome [[32], [33], [34]]. However, some studies, including a large-scale investigation [35], have failed to replicate the association between *AMY1*-CN and BMI, finding no correlation [35] or describing a diet-gene interaction [36], with the lowest BMI values reported among those with both low CN and high-starch intake, thus pointing to a low genetic capacity to metabolize digestible starches and partially break down some complex carbohydrates. The physiological and clinical effects of *AMY1* could also be mediated or synergistically enhanced by the composition of the microbiota, explaining the inconsistent findings reported in the literature [35]: in individuals with low *AMY1*-CN, *Prevotella* abundance in the gut and high *Prevotella*-to-*Bacteroides* ratios predict greater fat loss in response to plant-based dietary interventions, with more undigested starch reaching the colon and being processed by the gut microbiota [37,38]. Low *AMY1*-CN hosts have lower *Ruminococcus* and *Porphyromonas* abundance in the gut and oral microbiomes, respectively, than their high *AMY*-CN counterparts, thus harboring microbiota with enhanced capacity to break down complex carbohydrates and compensating for the reduced copies and activity of amylase [38,39].

However, it is debated when duplications of *AMY1* among humans and related molecular events took place. According to some researchers [30,40], these events would have been prompted by the transition to agriculture, whereas, according to others [41], they were induced by the adoption of cooking, with higher *AMY1-*CN providing the amount of glucose that the *Homo* lineage needed for their metabolically and biochemically demanding tasks.

Recently, Perry’s “starch digestion hypothesis” [30,40], suggesting that variations in the salivary amylase gene and its CNVs may play a significant role in starch digestion, with adaptive advantages, particularly for populations with high-starch diets, has been challenged [31,41,42], both physiologically (salivary α-amylase would play a limited role in starch metabolism) and genomically (higher *AMY1* copies may not confer any adaptive advantages). To the contrary, a relationship with the gene for pancreatic amylase (*AMY2A*) has been demonstrated in such a way that indirect evidence of phenotypic associations resulting from differences in the number of pancreatic amylase copies might manifest as subtle correlations with *AMY1*-CN [42] in terms of indirect effects or compensatory mechanisms within the body's overall strategy for starch metabolism. However, these contrasting results warrant further research, given that the role of *AMY1* and its relationship with health outcomes is still unclear.

## Introduction of Meat-Eating and Evolutionary Adaptation to Meat

The shift from primarily plant-based diets to a diet incorporating meat was facilitated by technologies, such as stone tools, which early hominins used for their scavenging activities. Stone tools provided several advantages, including the ability to cut, scrape, and process meat more efficiently. Pounding of food using hardwood or stones essentially acts as external mastication, offering new possibilities for the removal of antinutrients or facilitating fermentation. The drying of animal meat cut into thin slivers for future consumption represents a primeval form of food preservation enabled by lithic tools (in particular, sharp flakes obtained through knapping of large core hand axes), which prevented spoilage and extended meat shelf life, ensuring survival during periods of scarcity.

Increased consumption of energy- and nutrient-dense animal products could have provided essential dietary components further supporting brain development and growth. However, besides offering some benefits, the consumption of raw meat facilitated the spread of infectious agents while, at least partially, protecting against the development of hypercholesterolemia and cardiovascular disease that, instead, plague contemporary populations.

Similar to the heritability of the adaptations to plant consumption, the heritability of the adaptations to red meat consumption is also rather high (∼39%–44%) [28,29]. Genetic adaptations to meat-eating [[43], [44], [45], [46], [47]] would have occurred as adaptive mechanisms—the so-called “meat-adaptive genes” (MAGs), which became maladaptive in the last 100 y. The most notable MAG is apoE3, conferring a decreased risk of Alzheimer’s and cardiovascular disease in aging adults [46]. *MADD-FOLH1* is another MAG [45], whereas a subgroup of MAGs is involved in metabolic pathways, including cholesterol transport [47].

Other MAGs are linked to the buildup of the human immune system [48,49]. Immunological interactions with pathogens found in consumed meat began to alter hominins’ immunobiology, prompting a series of genomics events, including human-specific pseudogenizations (a series of processes by which functional genes lose their abilities to encode functional proteins, often due to mutations disrupting their expression or translation). One of these events targeted the *CMAH* gene. Through Alu-fusion–mediated loss of function, the mammalian sialic acid *N*-glycolylneuraminic acid was eliminated, generating an excess of its precursor *N*-acetylneuraminic acid. This changed the expression of ubiquitous cell surface “self-associated molecular patterns” that modulate innate immunity via engagement of CD33-related-*SIGLEC* receptors. These events resulted in the emergence of human-adapted pathogens with a marked preference for *N*-acetylneuraminic acid recognition and presenting *N*-acetylneuraminic acid-containing molecular mimics of human glycans that can suppress immune responses via CD33-related-*SIGLEC* engagement. Of note, high consumption of red meat is associated with increased cancer risk, in particular colorectal cancer. The formation of antibodies against the red meat-derived carbohydrate *N*-glycolylneuraminic acid has been proposed to play a role in carcinogenesis-related events [[50], [51], [52]].

Furthermore, meat consumption can result in an increased, microbiota-dependent cardiovascular disease risk: red meat is rich in *L*-carnitine, transformed by the gut microbiome into trimethylamine and metabolized by hepatic flavin-containing-monooxygenase-3 into trimethylamine-*N*-oxide (TMAO), which has been shown to favor atherosclerosis in murine models [53,54]. TMAO concentrations are higher in omnivorous humans than in vegans and vegetarians, and strong associations have been shown with coronary artery disease and neurodegenerative disorders [[53], [54], [55]].

Finally, meat diets may have left genetic marks in human taste receptor gene variants [56], with these adaptations shaping taste preferences, influencing dietary choices, and impacting human health in various ways. Understanding these genetic variations could provide valuable insights into human evolution, nutrition, and health.

## Control of Fire, Cooking, and Related Evolutionary Adaptations

The discovery and control of fire brought about profound changes in human dietary habits [57], as cooking is a highly effective method for food processing. It allows for the breakdown of complex carbohydrates and proteins, making them easier to digest, reducing diet-induced thermogenesis, increasing the bioavailability of some nutrients, and improving the energy gained from foods such as meat and plants [58]. Also, it helps eliminate harmful pathogens. To the contrary, cooking can accelerate the production of acrylamides linked to the insurgence of malignancies [59]. In response to the increased production of acrylamides, evolution has developed the aryl hydrocarbon receptor (AhR), a highly conserved, ligand-activated transcription factor involved in various signaling pathways and with significant effects on cell specialization, host defense, and detoxification [60].

Thermal treatment, as well as fermentation, have played crucial roles in human dietary habits, allowing the inactivation of dangerous or toxic antinutrients (lectins, protease inhibitors, saponins, phytates, tannins, glucosinolates, and cyanogenic glycosides, among others) in various food sources, particularly pulses and tubers. It is possible that the introduction of fire was preceded by geothermal cooking, which, by exploiting hydrothermal springs, may have allowed early hominins to cook plants and meat, increasing digestibility and nutritional value [61], even though documented evidence is scarce.

According to Miller–Colagiuri’s “carnivore connection” hypothesis [62,63], meat-eating and control of fire would have “prepared” the next dietary shifts. This theory posits that during periods of human evolution, particularly in the Paleolithic era, human ancestors who could efficiently store fat from the sporadic and protein-rich animal-based diet would have had a survival advantage during times of food scarcity. The necessity to transition from periods of limited availability of dietary carbohydrates in diets with a low ratio of plant- to animal-based subsistence, low carbohydrate intake, and low glycemic index to conditions characterized by high-carbohydrate diets would have selected positively for the genes of insulin resistance and have led to its development. In other terms, when the availability of fire made the consumption of meat more efficient while fewer dietary carbohydrates were consumed, individuals began to show increased gluconeogenesis and peripheral insulin resistance.

Even though these conditions usually imply the development of metabolic impairments, they could have offered survival and reproductive advantages during the 9 Ice Ages, which dominated most of human evolution. Those individuals with higher inherent insulin resistance were, indeed, able to shift glucose away from maternal utilization toward fetal metabolism, leading to an increase in birth weight and improved survival rates among offspring. The onset of the Agricultural Revolution marked a pivotal shift in human dietary patterns, primarily through the incorporation of large quantities of cereals, leading to a higher intake of carbohydrates and foods with a high glycemic index. This change impacted evolutionary selection pressures related to metabolism and dietary adaptations. Before agriculture, humans relied on a varied diet of hunted and foraged foods, which included a balance of proteins, fats, and carbohydrates. The ability to efficiently process and metabolize these foods was crucial for survival, imposing a strong selection pressure for metabolic efficiency in these conditions. However, with the introduction of agriculture and the subsequent increase in carbohydrate-rich cereals in the human diet, the selection pressure that favored certain metabolic traits began to ease.

Societies with a longer history of agriculture saw a gradual adaptation to these dietary changes, leading to a relaxation of the selection pressures that once favored traits necessary for a hunter-gatherer diet. In contrast, societies that faced geographic isolation experienced frequent periods of starvation and famines, or had shorter exposures to agricultural diets continued to experience intense selection pressures. These conditions would necessitate a more efficient metabolism of diverse and less predictable food sources, maintaining the selection for metabolic traits adapted to a variety of dietary conditions.

This evolutionary perspective offers insight into the rise of metabolic diseases throughout human history. Populations that rapidly transitioned to agricultural diets, such as the Pima Indians, experienced a sudden change in dietary intake without sufficient time for genetic adaptation, which likely contributed to a higher prevalence rate of metabolic diseases due to the mismatch between their genetic predispositions and the new dietary environment. To the contrary, populations in Europe, where the transition to agricultural diets occurred more gradually over thousands of years, had more time to genetically adapt to the changes in diet. This gradual transition could explain the lower prevalence of metabolic diseases in these populations than in those that experienced rapid dietary changes [64].

In summary, the shift to agriculture and the associated dietary changes may have led to a relaxation of selection pressures in societies with long agricultural histories, whereas those with shorter exposures or additional challenges faced intensified selection pressures and the evolutionary advantages of insulin resistance in a high-meat, low-carbohydrate environment have become disadvantages in today’s high-carbohydrate dietary landscapes. These complex dynamics have played a crucial role in the development of metabolic diseases, influenced by the rate and nature of dietary transitions across different human populations [64]. As we will see, in modern times, characterized by abundant food supplies, high-calorie foods, and high refined carbohydrate consumption, the genetic predisposition to insulin resistance has become maladaptive, leading to chronic high blood sugar, obesity, and type 2 diabetes.

Also, cooking of food components profoundly impacts the gut microbiome, both structurally and functionally [65]. More specifically, although microorganisms possess the innate ability to decompose resistant starches and plant-based fibers, humans have to rely on cooking, which facilitates the breakdown of these fibers. This allows for their byproducts to be directly absorbed in the small intestine without the intermediary action of microbes. In essence, cooking has facilitated a dietary shift toward the direct assimilation of numerous plant-based nutrients in the small intestine, reducing dependency on the metabolic processes of gut microbiota. Experiments [65] have shown, indeed, that consuming a plant-based diet in its raw form instead of cooked leads to a transformation in the gut microbiota of mice, driven by enhanced starch digestion and the breakdown of compounds originating from plants. These changes in the gut microbial community can influence the energy status of the host and apply to various starch-rich plants, findings that are also observable in humans. Hence, the influence of diet on the host is contingent on both the type and composition of the food and its preparation (“what and how we eat”) [66]. This interaction appears to be nutrient-/food-specific: although both food composition and cooking method seem to influence gut microbial community structure and function, with a release of short-chain fatty acids, intense cooking (roasting or grilling) results in an increased abundance of beneficial/healthy bacteria such as *Ruminococcus* and *Bifidobacterium* compared with milder cooking (boiling) for some foods, but not others (such as banana or bread) [66].

Moreover, as also previously said, eating raw, plant-based foods required a greater amount of microbial breakdown and fermentation in the distal parts of the intestine, resulting in a smaller amount of calorie absorption from the same amount of food, compared with the rapid absorption of cooked food in the proximal small intestine. The introduction of cooking would ultimately lead to a shortening of the large intestine and a reduced dependence on microbial symbionts, resulting in an increased availability of calories, which was adaptive for human ancestors but has become maladaptive in the last 100 y.

To the contrary, ethnohistoric literature shows that hunter-gatherers and traditional small-scale rural farmers regularly consumed putrefied meat, fish, and fat, generally raw and uncooked, without incurring the toxic effects of the metabolites of *Clostridium botulinum* and other pathogens. This could be due to environmental exposures to infectious agents through early childhood, which may have primed gut microbiota and immune systems, as well as to genetic factors, the pressure of which would have eased after the widespread habit of cooking foods, especially after European colonization, westernization, urbanization, and industrialization. Also, it should be noted that putrefaction “predigests” meat and fat prior to their ingestion, offering benefits comparable with cooking [67].

Nevertheless, the contribution of food processing to the modulation of gut microbiota diversity and functionality is still a matter of debate and requires further research.

## Plant and Animal Domestication and Evolutionary Adaptations

Marking the beginning of the Neolithic, the transition from a hunter-gatherer lifestyle to agriculture, dairying, and animal husbandry represents a major dietary shift accelerated by food processing, production, and storage technologies. Humans began cultivating crops and domesticating animals, leading to a more stable, predictable food supply, which enabled the growth of settled communities and laid the foundation for civilizations.

From a molecular perspective, the most notable signature of the transition to agriculture and pastoralism is given by lactase persistence [68,69], an example of niche construction, or an organism-induced modification of selective environments [70], which refers to the process (or series of processes) where organisms, through their activities and choices, alter their own and each other's environments, thereby influencing the selection pressures they are subject to. In other words, with the domestication of dairy animals and the subsequent inclusion of milk in the diet of adults, a significant evolutionary change occurred in some human populations. Individuals with mutations allowing continued production of lactase into adulthood had a nutritional advantage in these dairy-farming societies, leading to the selection of this trait.

Another molecular event that has been linked to the transition to agriculture is the insurgence of *NAT2* slow acetylator alleles: hunter-gatherer individuals were mostly fast acetylators, whereas agriculturalists are mainly slower acetylators, probably reflecting significant changes in lifestyles as well as in dietary exposure to environmental chemicals. Data from 128 population samples, totaling 14,679 individuals, found a significantly higher rate of the slow acetylation phenotype in populations practicing farming (45.4%) and herding (48.2%) than that in populations relying on hunting and gathering (22.4%). Similarly, the slow variant occurs at a 3-fold higher frequency in food producers than in hunter-gatherers (25% compared with 8%) [71,72]. However, evolutionary dietary adaptations come with a price, being initially advantageous and becoming maladaptive for modern humans: *NAT2* slow acetylator genotypes have an increased risk of developing autoimmune and chronic diseases and are more susceptible to drug side effects in that nonacetylated xenobiotics may accumulate and be metabolized by other enzymes into reactive intermediates.

Besides the *NAT2* pathway, other genetic signatures of the transition from “food collection” to “food production” include genes involved in lipid metabolism, glycolysis, folate pathway, and other micronutrient-related metabolic pathways [[73], [74], [75], [76]] (Table 2). They would have conferred several advantages, including improved digestion of starches and grains, enhanced fat metabolism, enhanced folate utilization, resistance to diet-related diseases, and increased reproductive fitness. Of particular importance is, indeed, the link between diet-related genes and the reproductive system [77]. Mutations of these genes are associated with a higher prevalence of chronic degenerative disorders, including malignancies, type 2 diabetes, diabetic nephropathy, and primary ovarian failure.

**TABLE 2:** A comprehensive overview of diet-related adaptive genes

Dietary exposure	Diet-related adaptive genes	Impact on health and disease
Exposure to plants and tough foods	*CYP* (cytochrome P-450) cluster, *ENAM* (enamelin), *SLC* (solute carrier family) cluster, *SLCO* (Solute Carrier Organic Anion Transporter Family Member) cluster	Salt-sensitive hypertension, metabolic diseases
Exposure to dietary aliphatic alcohols, including ethanol, through fermenting fruits and nectars; rice domestication	*ADH* (alcohol dehydrogenase) cluster, *ALDH2* (aldehyde dehydrogenase 2)	Drinking behavior and alcohol addiction, upper aerodigestive tract malignancies
Exposure to salt	*ADRB2* (β2 adrenergic receptor), *AGT* (angiotensinogen), *GNB3* (G protein b3 subunit)	Hypertension and cardiovascular issues
Exposure to starch	*AMY* cluster (*AMY1*, salivary alpha amylase)	Metabolic diseases, overweight and obesity, type 2 diabetes
Exposure to meat and fish	*APO* (apolipoprotein) cluster, *AGXT* (alanine:glyoxylate aminotransferase), *FADS* (fatty acid deacetylase) cluster (*FADS1*, *FADS2*), *MADD-FOLH1* (MAP-kinase activating death domain-folate hydrolase 1)*, MTHFR* (methylenetetrahydrofolate reductase), *MTRR* (methionine synthase reductase), *NAT2* (*N*-acetyltransferase 2 or arylamine *N*-acetyltransferase)	Alzheimer’s disease, chronic diseases, including rheumatoid arthritis, cardiovascular disease, and malignancies
Exposure to milk and dairy products	*LCT/CM6* (lactase)	Lactose intolerance

The human fatty acid desaturase (*FADS*) genes, clustering on chromosome 11, represent other genes selected during the phase of plant and animal domestication [[78], [79], [80]]. *FADS* are of interest in the context of adaptation to dietary changes because they may have played a role in the evolution of human diets, which varied based on the geographical locations and available food sources and, consequently, in terms of fatty acids. Genetic variants in *FADS1* and *FADS2* correlate with the variability of long-chain PUFA concentrations in serum and plasma, erythrocyte membranes, and adipocytes. Moreover, they have been associated with autoinflammatory diseases, cardiovascular disease, and type 2 diabetes [[73], [74], [75], [76]].

However, the hypothesis linking *FADS* with human adaptation to agriculture has been recently challenged. These genes were not strongly selected in European farming populations until probably the Bronze Age, whereas *AMY1*-CNV was selected much earlier [81]. As such, given the uncertainty and conflicting hypotheses, much research is needed.

Also, *FADS* have played a key role in the metabolism of PUFAs contained in aquatic foods, both freshwater and marine. The collection of mollusks, including both gastropods and bivalves, would have not only supplied valuable nutrients (essential fatty acids, iodine, and a variety of other micronutrients) but also exposed early humans to a variety of parasites. These mollusks also served as raw materials for symbolic behavior, such as ornamentation, and facilitated early trade networks. The coastal migrations of human ancestors out of Africa almost certainly benefited significantly from the abundance of shellfish and the relatively easy hunting of marine mammals such as seals, sea lions, and manatees. These resources would have provided a reliable and rich source of nutrition, supporting the survival and expansion of early human populations along coastal routes.

Finally, plant and animal domestication has fostered social activities, increased community sizes, and facilitated the spread of infections. Both evolutionary adaptations and environmental exposures have contributed to shaping the immune system and favored the insurgence of human-adapted foodborne pathogens [82,83], as well as a rich, diverse gut microbiome.

(Mal)adaptations to dietary shifts and the adoption of more sedentary lifestyles would have resulted in the emergence of metabolic disorders and cardiovascular disease—the so-called Neel’s “thrifty genotype” hypothesis [84], according to which body fatness would play a role in thermogenesis and cognition and would have conferred a survival advantage to human ancestors in the face of feast-famine cycles. However, according to other scholars, adiposity is not an adaptive trait: Speakman’s “predation release hypothesis” [85] posits that the genetic predisposition to obesity would arise due to the relaxation of predation pressures combined with random genetic drift.

## Introduction of Salt and Evolutionary Adaptations

The introduction of salt is another major dietary milestone in the history of human nutrition. In ancient times, when access to diverse sources of nutrients was limited, salt provided a concentrated source of these important minerals. Before the advent of modern refrigeration, salt was used as a preservative, besides containing minerals essential for maintaining fluid balance, nerve function, and muscle contractions. An added benefit of salt intake was its ability to enhance food flavor and palatability. However, although salt is essential for health, excessive consumption in the modern diet can lead to hypertension and related cardiovascular problems.

From an evolutionary standpoint, given “the duration of the gathering and hunting experience and our protracted existence in tropical biomes, an electrolyte-conserving phenotype” [86] was selected: genes related to blood pressure regulation and electrolyte flux have played a role in the response to dietary salt as well in energy restriction-induced adaptations [86,87]. Finally, the evolutionary history of these genes is multifaceted since out-of-Africa migration, expansion to new territories, and dramatical historical events, such as the trans-Atlantic Middle Passage, the forced voyage of enslaved Africans across the Atlantic Ocean to the New World, have contributed to adding further layers of complexity. The forced migration during the transatlantic slave trade and subsequent generations of living in often challenging environments could have contributed to the selection of certain genetic traits that now manifest as increased susceptibility to obesity and metabolic diseases in an environment that is markedly different from that of their ancestors and interact with a multitude of other factors, including socioeconomic status, cultural dietary practices, and access to healthy foods and safe spaces for physical activity. Similarly, a complex interplay of genetic, environmental, and historical factors can explain the high rates of obesity among indigenous populations around the globe, who historically had lifestyles that varied between periods of feast and famine and might have developed genetic predispositions to be particularly efficient at storing fat during times of plenty, to survive during times of scarcity (the “thrifty gene hypothesis” [84], mentioned earlier). However, with the drastic changes in lifestyle and food availability due to colonization, sedentarization, and globalization, these previously advantageous genetic traits may now predispose these populations to higher rates of obesity and related metabolic disorders.

## Industrialization, Modern Diets, and Evolutionary Adaptations

Characterized by urbanization, technological advancements, and shifts in labor and production methods, the Industrial Revolution brought about profound changes in dietary patterns and eating habits [[88], [89], [90]].

Nonchemical ways of food preservation such as cooking, refrigeration, fermentation, and addition of salt have been critical aspects of human food preparation, safety, and supply chain, as they can significantly extend its shelf life, prevent spoilage, maintain its nutritional quality, decrease microbial contamination events, reduce dependency on seasonality and geographic availability, mitigate risk of food shortages and famine, and favor migration and settlement in areas characterized by the availability of diverse foods.

Moreover, as people moved from rural areas to cities to work in factories and industries, their diets underwent substantial transformations, leading to adaptations, as they faced challenges obtaining fresh food due to limited space for agriculture and longer distances between farms and urban centers. There was a dramatic increase in processed and convenience foods, driven by the rise of industrialization, which facilitated the development of processed and preserved foods, such as canned goods and (pre-)packaged meals, with longer shelf life and more suitable for the fast-paced urban lifestyle. However, this led to a shift from nutrient-dense diets to less nutritious diets higher in refined carbohydrates, sugars, and less healthy fats, contributing to health issues such as malnutrition, obesity, metabolic diseases, and dental problems.

Some broad bacterial *taxa* (referred to as “volatile and/or associated negatively with industrialized societies of humans” *taxa*) are highly prevalent in Neolithic, Mesolithic, Middle Ages, and present-day indigenous populations living traditional lifestyles but are rare or absent in industrialized populations. Other bacterial *taxa* (referred to as “bloom or selected in societies of urbanization/modernization” *taxa*) show opposite patterns. The microbiota from nonindustrialized settings is more likely to show higher total microbial diversity, and research on human communities still adhering to traditional subsistence methods, largely untouched by the influences of urban and industrialized lifestyles, such as the Yanomami people, has confirmed such findings [91,92].

An analysis of the ancient microbial DNA in the calcified dental plaque from early European skeletons has shown a shift in the oral microbiome, reflecting the uptake of highly processed carbohydrate-rich diets, including flour and white sugar. Although the dental calculus of hunter-gatherer individuals had fewer microbial *taxa* related to periodontal diseases or caries, modern samples are dominated by cariogenic bacteria, with less oral microbial diversity. “The distribution of dental caries in patients aged >35 y can be superimposed to that of the countries” [93] where the food industry has developed. *Streptococcus mutans* represents the oral microbial signature of the Industrial Revolution [93,94], together with shifts in membership, composition/architecture, and function in human microbiota, resulting in “a configuration never before experienced by human populations.” This “new” industrial microbiota has been shaped by recent “progress in medicine” (including widespread use of antibiotics), “food, and sanitation” [88,89].

## The Postindustrial Period and Evolutionary Adaptations

With the widespread adoption of chemical agriculture practices, including the use of chemical fertilizers, synthetic pesticides, and insecticides to increase crop yields and protect them from pests and diseases, the postindustrial period, particularly the last 75 y since the end of World War II, has brought about significant changes in food production and preservation, from mass production techniques, such as factory farming, to antibiotic use and large-scale food processing, with a dramatic impact on the human gut microbiome and overall health.

The rise of the fast-food industry and the overconsumption of certain energy-dense unhealthy products with added sugars and high in salt and SFA have been linked to an epidemic of chronic noncontagious diseases. Low-grade systemic immune activation triggered by the maladaptive interactions between a diet-adapted microbiome and the ancient gut-based immune system has been proposed as shared pathophysiological mechanisms. Also, in recent years, the shift toward industrial foods has introduced a variety of “nonfood” molecules into the human diet, including polysaccharides such as carrageenans and other sulfated polygalactoses, which are commonly found in a range of modern foods, including many vegan products. Despite their potential functional benefits, there is growing evidence suggesting that they can induce inflammatory changes in the gut and other organs, contributing not only to the increased prevalence of inflammatory bowel disease [95] but also to other chronic diseases. In this sense, although additives are highly regulated and scientifically assessed, their contribution to human health in the very long term is not understood and represents a major research topic.

## Recent Dietary Trends: Globalization and Evolutionary Adaptations

**FIGURE 1:**
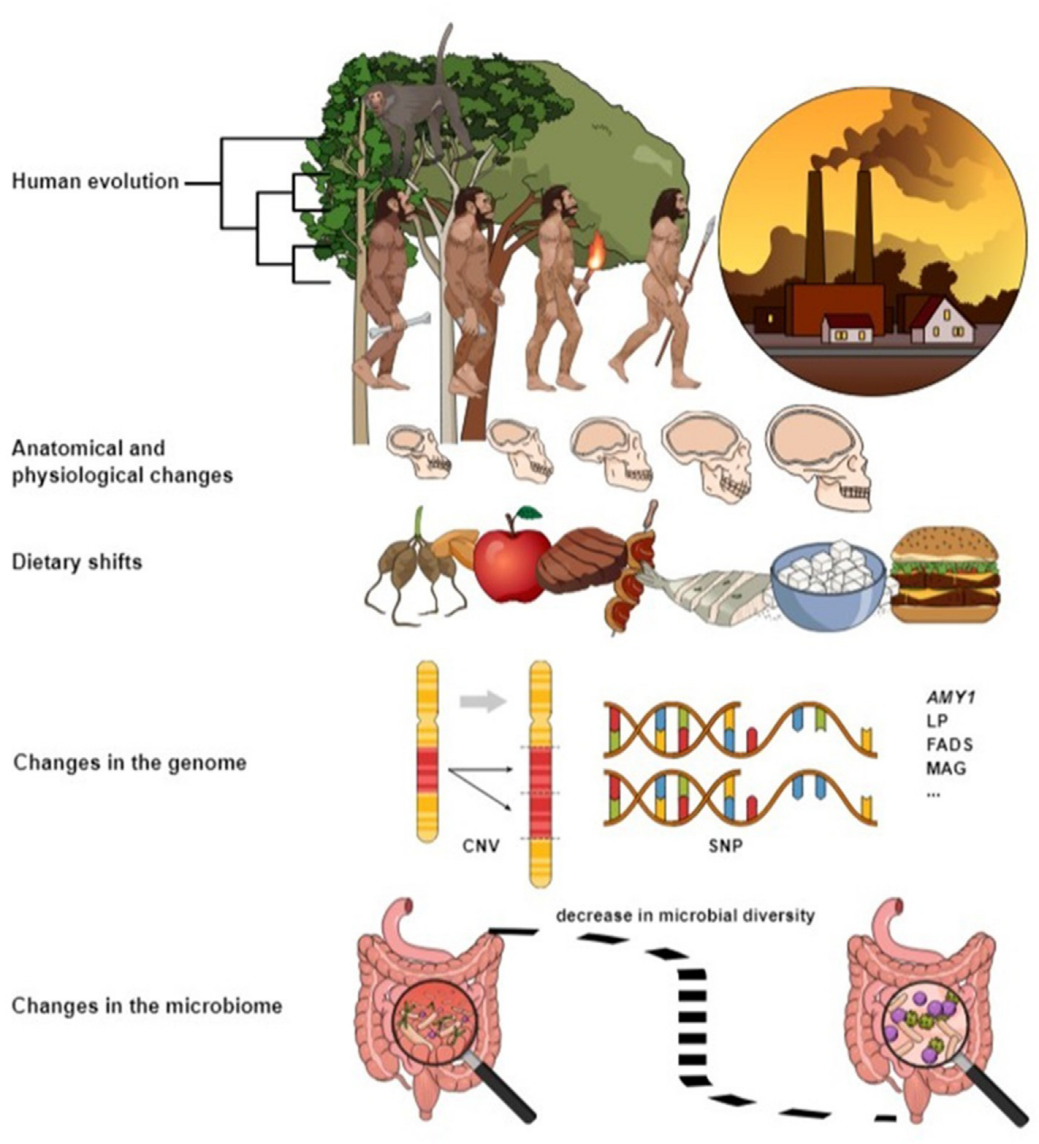
Pictorial diagram showing the major steps in human evolution, anatomical and physiological changes, dietary shifts, and changes in the human genome and microbiome.

Throughout human history, dietary habits have been shaped by a complex interplay and tradeoffs of biological, environmental, technological, and sociocultural factors: dietary and environmental exposures, coevolution, individual/genetic variability, microbiome, and gut health, as well as cultural, societal, and behavioral adaptations (Figure 1). Globalization-induced increased levels of stressors and “sources of perturbation such as sleep curtailment, reduced levels of physical activity and demanding mental work [that] have emerged from modernization” have resulted in a pandemic of noncommunicable diseases in the Western countries [88,89], whereas, in other parts of the world, human dietary choices have been and still are influenced by the availability of limited food resources. Climate change is anticipated to further exacerbate all this by reducing food availability, limiting food access, and impacting food quality.

Different diets can influence the composition and function of the gut microbiome, which, in turn, affects many physiological processes in the host, including nutrient absorption, metabolism, immune function, and overall health. Evolutionary changes in human diets have likely influenced the evolution of the gut microbiome, creating a mutually beneficial relationship between host and microbes: globalization has resulted in the “globalization of intestinal microbiota” [94], the implications of which on physical and mental health should be explored to devise and implement effective interventions.

### Future research directions

Scientific advancements have enabled the collection of unprecedented direct paleodietary details, even if the isolation of archaic food matrices remains analytically challenging. It can be anticipated that future technological breakouts and better integration of paleogenomics, paleoproteomics, paleoglycomics, and paleometabolomics [96,97] (Table 1) will allow the dissection of more relevant information concerning the diets of hominins and early human ancestors.

Assessing how ancestral diets supported health and survival and understanding the evolution and the impact of “diet-related adaptive genes” [98] can inform efforts to improve current dietary recommendations, address contemporary health challenges, and have far-reaching implications for human well-being and ecological footprint on the planet.

## Authors contributions

The authors’ responsibilities were as follows – NLB, DDR, EM, and PM: designed and wrote the manuscript; and all authors: read and approved the manuscript.

## Conflict of interest

The authors report no conflicts of interest.

## Funding

This work was supported by the National Recovery and Resilience Plan, Mission 4 Component 2 Investment 1.3 - Call for tender No. 341 of March 15, 2022, of Italian Ministry of University and Research funded by the European Union—NextGenerationEU; Award Number: Project code PE00000003, Concession Decree No. 1550 of October 11, 2022, adopted by the Italian Ministry of University and Research, CUP D93C22000890001, Project title “ON Foods - Research and innovation network on food and nutrition Sustainability, Safety and Security – Working ON Foods.”
